# Deletion and Down-Regulation of HRH4 Gene in Gastric Carcinomas: A Potential Correlation with Tumor Progression

**DOI:** 10.1371/journal.pone.0031207

**Published:** 2012-02-20

**Authors:** Chao Zhang, Yi Xiong, Jiana Li, Yang Yang, Li Liu, Wen Wang, Luo Wang, Manhui Li, Zhengyu Fang

**Affiliations:** Biomedical Research Institute, Shenzhen-PKU-HKUST Medical Center, Guangdong Province, Shenzhen, People's Republic of China; Baylor University Medical Center, United States of America

## Abstract

**Background:**

Histamine is an established growth factor for gastrointestinal malignancies. The effect of histamine is largely determined locally by the histamine receptor expression pattern. Histamine receptor H4 (HRH4), the newest member of the histamine receptor family, is positively expressed on the epithelium of the gastrointestinal tract, and its function remains to be elucidated. Previously, we reported the decreased expression of HRH4 in colorectal cancers and revealed its correlation with tumor proliferation. In the current study, we aimed to investigate the abnormalities of HRH4 gene in gastric carcinomas (GCs).

**Methodology/Principal Findings:**

We analyzed H4R expression in collected GC samples by quantitative PCR, Western blot analysis, and immunostaining. Our results showed that the protein and mRNA levels of HRH4 were reduced in some GC samples, especially in advanced GC samples. Copy number decrease of HRH4 gene was observed (17.6%, 23 out of 131), which was closely correlated with the attenuated expression of H4R. *In vitro* studies, using gastric cancer cell lines, showed that the alteration of HRH4 expression on gastric cancer cells influences tumor growth upon exposure to histamine.

**Conclusions/Significance:**

We show for the first time that deletion of HRH4 gene is present in GC cases and is closely correlated with attenuated gene expression. Down-regulation of HRH4 in gastric carcinomas plays a role in histamine-mediated growth control of GC cells.

## Introduction

Histamine is a ubiquitous chemical messenger that has been demonstrated to be involved in cell proliferation, embryonic development, and tumor growth. These various biological effects are mediated through the activation of specific histamine receptors (H1, H2, H3 and H4) that differ in their tissue expression patterns and functions [1]. Through these pharmacologically distinct receptors, histamine may act as an autocrine or paracrine growth factor that increases proliferation rate in malignant tissues [2], [3], [4]. Among the histamine receptor family, histamine receptor H1 (HRH1) and histamine receptor H2 (HRH2) have long been considered to be involved in histamine-mediated gastrointestinal cancer growth [5], [6], [7], [8]. Antagonists of HRH1 or HRH2 have been reported to be involved in the growth control of several types of tumors [1], [6], [9] and their inclusion in human therapy has been proposed.

The histamine receptor H4 (HRH4) is the most recently discovered histamine receptor and has a distinct pharmacological profile [10]. It plays a role in immunological and inflammatory processes and is predominantly expressed on hematopoietic and immune cells [11]. Quite recently, accumulated evidence indicates that HRH4 also plays a role in cell proliferation, both in normal and malignant cells, including hematopoietic progenitor cells [12], breast cancer cells [13], and pancreatic carcinoma cells [14].

HRH4 is positively expressed in the whole gastrointestinal tract [15], although its function remains unclear. Abnormalities of H4R in colorectal malignancies [16], [17] have been reported and the role of HRH4 has been postulated in the proliferation of colon carcinoma cells [17], [18]. Until now, however, little is known whether there are any abnormalities of HRH4 gene in gastric carcinomas (GCs).

GC is currently the most common cancer in China, responsible for about 300,000 deaths per year. Both genetic and environmental factors contribute to disease etiology. Studies using array comparative genomic hybridization (aCGH) have suggested that DNA deletions at chromosome position 18q11, also the chromosome locus of HRH4, are frequent in gastrointestinal malignancies [19], [20], [21], [22]. Here we aimed to examine the mRNA expression levels as well as copy number variations of HRH4 in a relatively larger number of GC samples.

Most of the aCGH experiments focused on the genome-wide screening of copy number variations (CNVs) and the data obtained are generally informative but not definitive. Thus, a study comprehensively examining CNVs in relation to HRH4 expression or prognosis should be performed using a large number of tumors. In this study, our approach was based on real-time PCR analysis, an established quantitative method examining the copy number and expression level of the targeted gene [23], [24]. Fluorescence in situ hybridization method was used to confirm the copy number variations (CNVs) of the HRH4 gene in the GCs. In addition, *in vitro* studies using GC cell line were carried out to reveal the potential role of HRH4 abnormalities in the progression of GC.

## Materials and Methods

### Patients and Tissue Collection

Gastric cancer samples were obtained from 131 surgical patients of the Department of Gastroenterology, Shenzhen Hospital, Peking University. A detailed description of patient characteristics was included in (Table S1). The GC tissues were collected from patients undergoing gastrectomy. Case-matched adjacent normal mucosa, located at least 2 cm far from the macroscopically unaffected margins of the tumor, were defined as normal controls. All the samples were stored in liquid nitrogen immediately after the operations. All 131 tumors were adenocarcinomas and other types of tumors (squamous cell, carcinoid, and stromal tumors) were excluded. Gastric carcinomas were staged according to the TNM classification system and graded into 5 groups: Stage0 (T_is_N_0_M_0_, n = 0), Stage1 (T_1_N_0–1_M_0_, n = 4), Stage2 (T_1_N_2_M_0_, T_2_N_0–1_M_0_, n = 30), Stage3 (T_2_N_2_M_0_, T_3_N_1–2_M_0_, T_4_N_0_M_0_, n = 54), Stage4 (T_4_N_1–2_M_0_, T_1–3_N_3_M_0_, T_any_N_any_M_1_, n = 43). The samples of Stage0–1 were combined into Stage2 due to the small sample size. Matched samples of GCs (n = 131) and adjacent normal tissues (ANTs, n = 131) were immediately stored in liquid nitrogen after operation. For each sample, a portion of tissue was disposed to make frozen sections and then micro-dissected by the Eppendorf Microdissection System (Siskiyou MX160L micromanipulator and PixeLink PL-A662 color CCD firewire camera). Then the dissected tissues were subjected to real-time PCR analysis. All patients were informed about the aims of specimen collection and gave signed written consent in accordance with the ethical guidelines of Peking University. Peripheral blood samples from 152 healthy controls were collected at Peking University People's Hospital. The investigations were conducted according to the Declaration of Helsinki principles and the study was approved by the ethical committee of Peking University Shenzhen Hospital.

### Western blotting

Tissue samples (after freezing and grinding) or cells were washed with PBS and lysed in a buffer containing 50 mM Tris-HCl (pH 6.8), 2% SDS, 10% glycerol, phosphatase inhibitors (100 mM Na_3_VO_4_, 10 mM NaF) and protease inhibitor (1 mM PMSF). Equal amounts of protein were loaded on a SDS–PAGE and transferred to PVDF membrane. After blocking with 5% non-fat milk in TBS-T (containing 0.1% Tween-20), the membranes were incubated with specific primary antibodies, followed by HRP-conjugated secondary antibodies. Proteins were visualized by fluorography using an enhanced chemiluminescence system. Data of the blotting were analyzed by the Total-Lab software (V2.01). Anti-HRH4 antibody was purchased from Chemicon International (Millipore, USA Canada); anti-cyclin D1, anti-p21 and anti-p27 antibodies were purchased from Cell Signaling Technology, Inc. (USA); anti-CDK2 and anti-αTubulin antibodies were purchased from Sigma-Aldrich Corporation (JPN).

### RT-PCR and Real-time quantitative PCR

Total RNA was isolated using the Trizol system according to the manufacturer's guidelines. Oligo (dT) 18 primer and M-MLV reverse transcriptase were used for first strand synthesis. The cDNA was then used as a template for real-time PCR and RT-PCR with gene specific primers. Real-time PCR was performed with Real-time PCR Master Mix containing SYBR GREEN I and hotstart Taq DNApolymerase. GAPDH was amplified as control. The primers for HRH4 and GAPDH are: HRH4 (sense): 5′- GTG GTT AGC ATA GGT TAT AC-3′, HRH4 (antisense): ′- ATG CCA CTG CAC TCC TGC-′; hGAPDH (Sense): 5′- CAGCCTCAAGATCATCAGCA-3′; hGAPDH (anti-sense):5′- TGTGGTCATGAGTCCTTC CA-3′ [24]. Real-time detection of the emission intensity of SYBR GREEN bound to double-stranded DNAs was performed using the I-cycler Instrument (Bio-rad). At the end point of PCR cycles, melt curves were made to check product purity. The level of H4R mRNA was expressed as a ratio relative to the GAPDH mRNA in each sample.

### Immunofluorescence staining

We selected a number of GC samples with significantly attenuated HRH4 expression (according to results from Western blot, n = 15) to make paraffin-embedded tissue sections (4 µm thick). Then the immunofluorescence staining was performed as described previously [25]. The anti-HRH4- antibody (1∶50, CHEMICON, US) and the secondary antibody, conjugated with CY3, (Jackson Immuno Research, US) were applied in the current study.

### DNA extraction and quantification of copy numbers

Genomic DNA was isolated from the tissues using the Genomic DNA Extraction Kit (Innocent, Shenzhen, China) according to the manufacturer's instruction. The concentration of purified DNA was determined by measuring the absorbance at 260 nm and 280 nm in a spectrophotometer. Pure preparations of DNA have OD260/OD280>1.8. Quantitative PCR was performed through BioRad Chromo4 real-time PCR system. The primers for RNAse P are: forward: 5′-AGA CTA GGG TCA GAA GCA A-3′ and reverse: 5′-CAT TTC ACT GAA TCC GTT C-3′.Two primer sets for HRH4 gene are applied in this study: (1) forward: 5′- GTG GTT AGC ATA GGT TAT AC′ and reverse: 5′- ATG CCA CTG CAC TCC TGC -3′ [24]; (2) forward: 5′-CAG AGA TAG GGC GAA GGA TT -3′ and reverse: 5′-TCC AAG CTC ACT CAC CCT TA-3′. PCR conditions for these reactions were: 95°C 5 min followed by 95°C 30 s, 57°C 25 s, 72°C 30 s (plate read) for 40cyles, then 72°C for 10 min.

Average copy numbers of RNAse P in normal candidates (copy numbers = 2) were used as control [24], [26]. The copy numbers of HRH4 were calculated by using the comparative Ct method. Each Ct value was obtained from two independent reactions. Cut-off values of 0.25, 0.75, 1.25, and 1.75 were used to define the copy numbers as 0, 1, 2, and 3, respectively. Fold change of each sample was presented as follows: fold change = relative expression level / average expression level in the group with 2 copies of DNA. A representative diagram for the DNA CNV analysis of the target gene in three GC/ANT pair was shown in Figure S1.

A standard curve was prepared using 2 µl of crude DNA solutions, in which serially diluted samples (original, 2-, 4-, 8-, 16-diluted) were included. The slopes of Ct and efficiency of each primer were calculated by the BioRad Chromo4 real-time PCR system and Microsoft Excel 2007 for Windows. Relative quantification of HRH4 was performed with the 2^−ΔΔCt^ method.

### Fluorescence in situ hybridization (FISH) analysis

GC tissues and matched adjacent normal tissues were prepared as introduced in the Materials and Methods. Ten N/T pairs (5 with HRH4 deletion, 5 with unaltered HRH4 copy number) were selected for FISH analysis. For isolation of cells, 0.075 m KCl was added to tissue for 10 min, and the tissue was minced with a scalpel to form a suspension of single cells. The cell suspension was fixed in methanol∶ acetic acid (3∶1). The suspension of isolated fixed nuclei was dropped on a slide and fixed by 70°C steam. Target slides were denatured in 2× SSC/70% formamide, pH 7, at 75°C for 5 min and dehydrated in graded ethanol.

Dual labeling hybridization with 10 µl of hybridization mix and a directly labeled centromere probe specific for chromosome 18q (Spectrum Green-labeled) along with a Spectrum Orange-labeled probe for the HRH4 locus (18q11) were performed. Probes were denatured at 75°C for 5 min and applied to the target slides. Hybridization was performed overnight at 37°C. Post-hybridization washes were performed with 50% formamide/2× SSC for 10 min three times, 2× SSC for 5 min, and 2× SSC/NP40 for 5 min at 45°C. Counterstaining was performed with 4′,6-diamino-2-phenylindole dihydrochloride. The number of FISH signals was counted with an Olympus microscope equipped with a triple band pass filter. A minimum of 300 nuclei were evaluated. FISH signals were counted according to the criteria described previously and were recorded as 0, 1, 2, 3, 4, 5, or more signals for each probe.

### Cells and culture conditions

The human gastric cancer cell lines (including AGS, MGC803, BGC823, HGC-27, SGC7901 and MKN-45) were obtained from the Department of Biochemistry, Hong Kong University of Science and Technology. The AGS line, whose characteristics of which are described in detail elsewhere [27], [28], were propagated in Dulbecco's modified Eagle's medium (DMEM, Gibco), supplemented with 10% fetal bovine serum (PAA) and 1% penicillin/streptomycin (Life Technologies, Inc.).

### Plasmids and transfection

We used a previously described [17] expression construct encoding HRH4 cDNA fragment (named pcDNA3-HRH4). To produce stable transfectants, pcDNA3, pcDNA-HRH4 plasmids were stably transfected to AGS line using Lipofectamine 2000 reagent (LF2000, Invitrogen, Carlsbad, CA) according to the manufacturer's recommendations. Selection was performed via the addition of 1.2 mg/ml G418. The transfectants from backbone vector and pcDNA3-HRH4 were designated as Mock-AGS and H4R-AGS, respectively

### Cell Proliferation and Cologenic Assay

Cell proliferation was measured by WST-1 assay. Mock-AGS and H4R-AGS cells were plated in 96-well culture plates (1×10^4^ per well) and treated with or without histamine (10^−5^ M). WST-1 (Roche) assay measuring the activity of mitochondrial dehydrogenases was performed following the manufacturer's instruction at 0-, 1-, 2-, 3- ,4- ,5- day time points.

To determine long-term effects, cologenic assay was used to elucidate the possible differences in long-term effects of altered HRH4 expression on human gastric cancer cells. Mock-AGS and H4R-AGS cells were trypsinized and counted using a hemocytometer. Cells (2×10^4^) were plated in the 6-well dishes and supplemented with histamine or clobenpropit 24 h later. Two weeks after the onset of drug selection, the cells were fixed and stained with crystal violet (0.1% crystal violet in 20% methanol). A cluster of a minimum of 50 cells is considered a colony.

### Flow cytometric analysis of cell cycle

For cell-cycle assay, cells were trypsinized with 2 mM EDTA in PBS and rinsed twice with ice-cold PBS solution, then fixed by adding them drop-wise into 75% ice-cold ethanol while vortexing, followed by incubation in ice for 60 min. The fixed cells were washed with ice-cold PBS and incubated at 37°C for 30 min in 0.5 ml PBS solution containing 20 µg/ml RNaseA, 0.2% Triton X-100, 0.2 mM EDTA and 20 µg/ml of propidium iodide. The percentage of cells in G0/G1, S, and G2/M phases was determined using the EPICS-XL flow cytometer (Beckman-Coulter, USA) and the Multicycler program.

### Statistical analysis

Statistical analysis was performed with the SPSS Software (version 12). Data of HRH4 copy number were analyzed by the chi-square test or Fisher exact test. P-values less than 0.05 were considered statistically significant. Results of the H4R mRNA expression for normal and tumor tissue samples were compared using two-way repeated measurement ANOVA. One-way, repeated measures analysis of variance (ANOVA-RM) was performed at a significance level of p = 0.05 to determine differences from controls within each group. Two-way analysis of variance (ANOVA-2) was performed after baseline subtraction, at a significance level of p = 0.05, to determine differences between the groups with deleted and unaltered HRH4 copy number. Statistical analysis of the results from flow cytrometry and colony forming assay were done with t-test, with statistical significance set at P<0.05.

## Results

### Attenuated expression levels HRH4 were observed in gastric adenocarcinomas and were correlated with tumor progression

**Figure 1:**
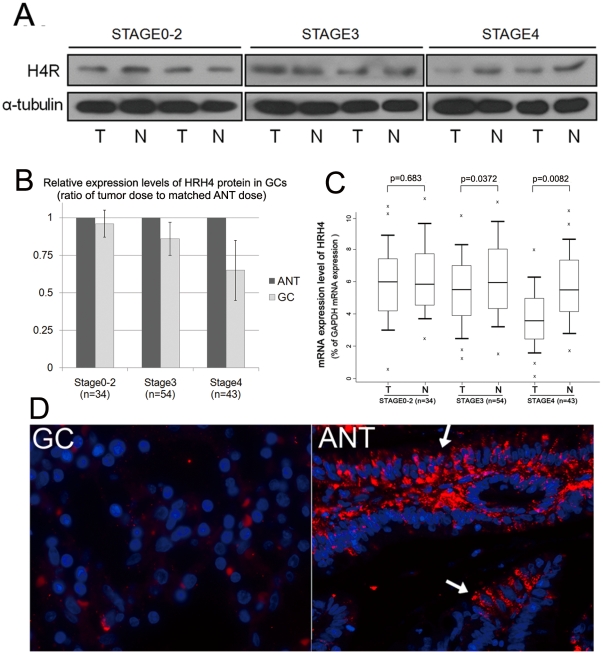
Decreased H4R expression in GCs. (A) Representative blots of HRH4 expression in normal mucosa and gastric tumor tissues. α-tubulin was used as a stable endogenous control. (B) The histogram shows the anlysis of the results from the immunoblottings. The relative expression value of HRH4 protein (normalized by α-tubulin) in GCs is expressed as an average ratio±s.e. of tumor dose to matched adjacent normal tissue dose. *p<0.01 vs. Control, ANT. (C) Real-time PCR assay was carried out as described under Materials and Methods section, boxplots of relative HRH4 mRNA(HRH4/GAPDH) measured with real-time PCR analysis showing median; box: 25th–75th percentile; bars: largest and smallest values within 1.5 box lengths; little cross: outliers. The results were obtained from 3 reactions in each sample. (D) Representative immunofluorescent microscope analysis of paired samples of GC tissue and adjacent normal tissue using anti human HRH4 monoclonal antibody (red). Nuclei were stain with DAPI (blue). Sample I: gastric cancer (GC); sample II: adjacent normal control (ANT). Arrows point to region of positive staining.

We first examined the expression levels of H4R in the collected GC samples. As shown in Figure 1A, attenuated expression levels of the HRH4 protein were mainly observed in advanced GC samples compared to matched adjacent normal tissues (ANTs).

Then we assessed the HRH4 mRNA expression in each T/N pair using quantitative PCR method. It was found that mRNA levels of HRH4 were reduced in the GC samples compared to ANTs (Figure 1B). There was a statistical difference between the group of early-stage GCs and advanced GCs. Figure 1C showed the representative immunocytochemistry analysis of HRH4 expression in selected GC samples. The obtained results were in accordance with the data from the immunoblottings.

### Copy number loss of HRH4 gene contributes to the gene repression in GCs

Little is known about the regulation of HRH4 expression. Since no detectable CpG island was found on the proximal region of HRH4 promoter (2 kbp, Figure S2), DNA methylation might not play an important role in this progress. DNA deletion at chromosome position 18q11, also the chromosome locus that HRH4 gene resides, is frequent in gastrointestinal cancers [20], [22]. We wondered if copy number variations (CNVs) of HRH4 gene might play a role in the regulation of gene expression.

We examined the copy number of HRH4 gene in each N/T pair as well as in healthy normal controls (HNCs). No statistical differences of copy number distribution between adjacent normal tissues (ANTs) and healthy normal controls were observed (Table 1). Thus, the ANT could be used as controls for the GC tissues in each N/T pair. As shown in Table 2, deletion of HRH4 gene was observed in a certain percentage (17.6%, 23 out of 131) of collected GC samples. Much higher frequency of HRH4 gene deletion was observed in the advanced GCs (21.6% for stage3–4) than those in early-stage GCs (5.88% for stage0–2).

**Table 1:** Comparison of CNVs of HRH4 gene between adjacent normal tissues (ANT) and healthy normal controls (HNC) * from peripheral blood.

Samples		Copy number					
	n	Deletion			Amplification		*p* (vs. HNC)
		0	1	2	3	>3	
HNC	152	1	3	144	4	0	---
ANT	131	3	4	121	2	1	0.989

*ANT represents adjacent normal tissues; HNC represents healthy normal controls

**Table 2:** CNVs of HRH4 in GC tissues and matched adjacent normal tissues (ANTs).

			Copy numbers		P (vs. ANT)	P (vs. Stage0-2)
CNVs, population		n		Deletion		
			<$>\raster="rg1"<$>2	<2		
Total	ANT	131	127	4	---	---
	GC		108	23	1.13E-04	---
Stage0-2	ANT	34	33	1	---	---
	GC		32	2	5.54E-01	---
Stage3	ANT	54	52	2	---	---
	GC		44	10	1.09E-02	9.25E-2
Stage4	ANT	43	42	1	---	---

**Figure 2:**
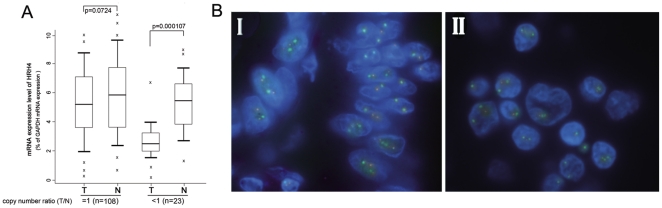
Copy number loss of HRH4 in GCs. (A) Real-time PCR assay was carried out as described under Materials and Methods section, and the results were obtained from indicated group of samples. Boxplots of relative copy number of HRH4 mRNA measured with Real-Time PCR analysis showing median; box: 25th–75th percentile; bars: largest and smallest values within 1.5 box lengths; little cross: outliers. mRNA expression level of HRH4 in groups with deleted (n = 23) or unaltered (n = 108) DNA copies. (B) Representative figure of FISH analysis using chromosome 18q specific alpha satellite DNA probe and chromosome 18q11 specific probe for HRH4 gene. I. Nucleus of ANT tissue with two signals for each of green and orange, showing no deletion of chromosome 18q or HRH4 gene. II. Nucleus of GC tissue with 0–2 signals for green and 0–2 signals for orange, indicating relative deletion in chromosome 18q or HRH4 gene.

It is expected that the CNVs do have genotype-phenotype correlation. Next, we selected the samples with either decreased or unaltered copies of HRH4 and tested whether the HRH4 mRNA expression was correlated with the copy numbers. As shown in Figure 2A, the mRNA level in the group with deleted copies of HRH4 was significantly lower than those with unaltered copies (p = 0.000109). Thus, the copy number loss of HRH4 at least plays a role in the down-regulation of HRH4 expression in GCs. On the other hand, the reduced HRH4 expression was also observed in the GC samples with unaltered copies, which indicated that there are other mechanisms involved as well.

To confirm the CNVs of HRH4 gene in GCs, fluorescence *in situ* hybridization analysis of chromosome 18q and 18q11 specific probe (HRH4) were also performed in selected paraffin-embedded GC tissues and matched ANT tissues (n = 10, 5 deletion, 5 unaltered). The results obtained were in accordance with those from real-time PCR analysis (Figure 2B and Table 3).

**Table 3:** FISH results in 10 GC/ANT pairs.

Results from Real-time PCR analysis	Case number	Stage	GC		ANT		HRH4 gene deletion Ratio (T/N)
			Deletion of 18q11 (HRH4)	Deletion of 18q	Deletion of 18q11 (HRH4)	Deletion of 18q	
Unaltered HRH4 gene copy number	1	2	−	−	−	−	0.97
	2	3	−	−	−	−	1.03
	3	3	−	−	−	−	0.92
	4	3	−	−	−	−	1.15
	5	4	−	−	−	−	1.02
HRH4 gene deletion	6	2	+	−	−	−	0.39
	7	3	+	−	−	+	0.71
	8	4	+	+	−	−	0.44
	9	4	+	−	−	−	0.67
	10	4	+	+	−	−	0.19

### Alteration of HRH4 expression influences histamine-mediated cell growth in gastric cancer cells

To investigate the role of impaired HRH4 expression in gastric cancer, *in vitro* experiments using gastric cell lines were performed. We first assessed the expression of HRH4 in several gastric cancer cell lines (Figure S3). The AGS line with relatively lower expression of H4R was selected. FISH approaches revealed the loss of heterozygosity (LOH) of HRH4 gene in this cell line (Figure S4).

**Figure 3:**
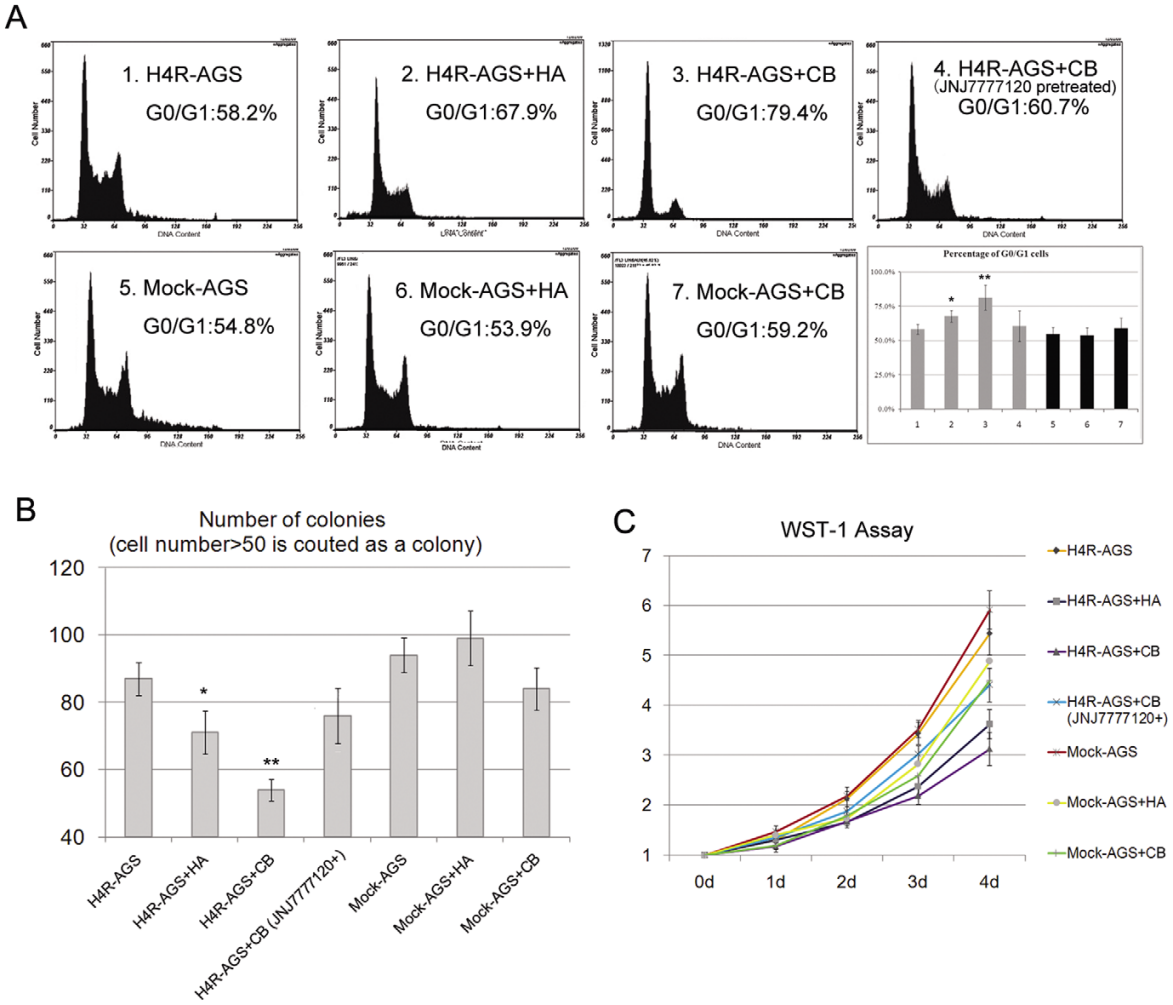
HRH4 activation induced growth arrest in gastric carcinoma cell lines. (A) Mock-AGS and H4R-AGS cells were treated with 10^−5^ M histamine, CB or CB accompanied by HRH4 antagonist (JNJ7777120) pretreatment, and cell-cycle distributions were determined by propidium iodide flow cytometry analysis. Each value is the mean±s.e. of triplicate data representative for three independent experiments. *p<0.05 and **p<0.01 vs. Control, H4R-AGS cells without any treatment. (B) Colony-formation assay. 5×10^3^ Mock-AGS and H4R-AGS cells were treated as in Fig. 3A and maintained in G418 for 14 days, and the colonies were stained with Giemsa. The bar graph shows the absolute colony (≥50 cells) number±s.e. in duplicate experiments. *p<0.05 and **p<0.01 vs. Control, H4R-AGS cells without any treatment. (C) Mock-AGS and H4R-AGS cells were treated as in Fig. 3A, WST-1 (Roche) assay measuring the activity of mitochondrial dehydrogenases was performed following the manufacturer's instruction at 0-, 1-, 2-, 3- ,4- ,5- day time points. Each value is the means.e. of triplicate data representative for three independent experiments. Error bars represent standard deviation of the mean.

H4R has been indicated to be involved in cell growth control in different types of cells [12], [13], [14]. We wondered whether altered expression of H4R could influence the proliferational ability of the GC cells from exposure to histamine. We used histamine (HA), the natural ligand of H4R, and clobenpropit (CB), one of the most specific agonist of H4R, to activate H4R. Although CB is capable of activating both H3R and H4R, it is an HRH4-specific agonist here because gastric epithelium seldom express H3R [15]. As shown in Figure 3A, both histamine and CB inhibited proliferation of H4R-AGS cells at an optimal dose of 10^−5^ M, as illustrated by the accumulation of cells in stage G0/G1 and decrease in G2/S, while CB treatment has little influence on Mock-AGS cells. Moreover, pre-treatment with the selective HRH4 antagonist JNJ7777120 before exposure to histamine or CB prevented the cell cycle arrest, providing additional evidence of the involvement of H4R in the regulation of cell cycle.

To evaluate the long-term tumor growth influenced by alteration of H4R expression, we performed the colony-forming assay. As shown in Figure 3B, cells transfected with the HRH4 gene generated a lower number of colonies than those transfected with the backbone vector after histamine treatment. Moreover, we analyzed the growth potential of H4R-AGS cells by WST-1 assay at different times after plating. The H4R-AGS cells grew at a significantly slower rate when incubated with medium containing histamine, while growth of Mock-AGS cells was not influenced by the natural ligand of H4R (Figure 3C).

### Effects of HRH4 activation on cell-cycle regulatory molecules in GC cells

**Figure 4:**
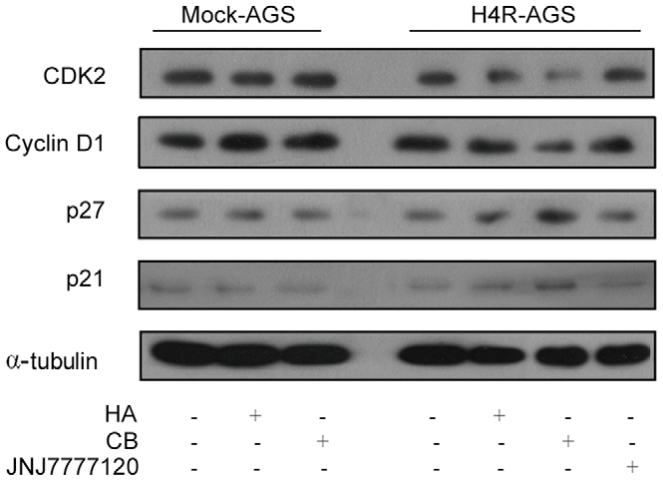
Expressions of cell cycle proteins regulated by HRH4. Mock-AGS and H4R-AGS were treated as in Fig. 3A, and total cell lysates were examined by western blotting.

The CB-induced G1 arrest was further confirmed by examining the cellular levels of the G1 cell-cycle control proteins cyclin D1 and Cdk2 in H4R-AGS cells. After H4R-AGS cells were treated with 10^−5^ M CB or HA, the expression levels of cyclin D1 and Cdk2 were attenuated while the expression levels of p21^Cip1^ and p27^Kip1^ were substantially up-regulated stimulated by CB after 24 h. (Fig. 4A). Based on these findings, CB-mediated activation of HRH4 is likely to block the cell-cycle progression through G1 to S phase. Expression levels of cycle proteins in Mock-AGS cells were not significantly influenced by CB or histamine treatment, which further confirmed the role of HRH4 in this progress.

## Discussion

The distribution of H1R, H2R and H4R in the epithelium of gastrointestinal tissue has been indicated [15]. The relevance of HRH4 abnormalities in colorectal cancer has been reported by different groups [16], [17]. In the current study, we first showed that abnormalities of H4R were also present in GC tissues, which was a novel finding. In colorectal cancers, expression levels of HRH4 mRNA were reduced regardless of the grade or Dukes classification of the tumors [16], [17]. In gastric malignancies, however, attenuated H4R expression was mainly observed in advanced GCs, which suggested that deletion and down-regulation of HRH4 might mainly take place in the progression but not the initiation of GCs.

The involvement of copy number variation (CNV) in the regulation of HRH4 expression in GCs was also a novel finding. Chromosomal aberrations such as deletions, amplifications, and structural rearrangements are hall markers of somatic cancer and lead to DNA copy number alterations with associated gain or loss of genes important for tumor progression. CNVs identified by CGH and array technology have been clearly shown to have the potential to directly or indirectly influence a healthy individual's susceptibility to cancer, for example by varying the gene dosage of tumor suppressors or oncogenes [29]. However, there are many discrepancies among previous studies which used high-resolution aCGH approaches to screen CNV. In the current study, the real-time quantitative PCR method was applied to analyze the copy number change in a relatively larger number of GC/ANT pairs, which could alleviate the risk of false positives brought by aCGH. Additional experiments using FISH analysis were also performed to validate the obtained data. These helped to increase the reliability of the results.

Since there were no statistical differences of CNVs of HRH4 between blood samples from healthy normal controls and adjacent normal tissues from GC patients, the CNVs of HRH4 in GCs were more likely acquired DNA aberrations. There was a significant correlation between copy number deletion and down-regulation of HRH4 gene in GCs, while the expression level of HRH4 mRNA was also impaired in the group of GC samples with unaltered copy numbers. Thus, there were different mechanisms involved in the regulation of H4R expression in GCs, which warrants further investigation.

In the histamine receptor family, HRH2 has long been known to be important in both acid secretion and growth and differentiation of the gastric mucosa [30]. Antagonist of H2R could alter the proliferation level of gastric cancer [31], [32]. As for HRH4, no functional elucidation in gastric enterocytes has been described. In this study, the results from the *in vitro* approaches using GC cell line suggested that HRH4 stimulation could cause cell growth arrest through the induction of the cyclin-dependent kinase inhibitors, including p21^Cip1^ and p27^Kip1^. p21^Cip1^ and p27^Kip1^ play important roles in mediating growth arrest and are considered to function as brakes of the cell cycle [33]. The increase in p21^Cip1^ and p27^Kip1^ could, by inhibiting cyclin D1/Cdk4 or Cdk6 kinase activity, explain, at least partially, the increase of cells in the G1 phase by CB treatment in our study.

Overall, our results first revealed the presence of the abnormalities of HRH4 in GCs and suggested its potential role in histamine-mediated regulation of tumor growth. However, the *in vivo* study underlying mechanisms of the HRH4 expression levels, using HRH4-knockout mice, needs to be investigated in the future.

## Supporting Information

Figure S1 **The representative diagram of the DNA CNV analysis for oneT/N pair.** (A) Real-time PCR amplification of targeted gene in selected genomic DNA samples. Each data was obtained from two independent reactions. (B) Original Ct values obtained from the real-time PCR amplification. (C&D) The efficiency of and slope of the RNAse P and HRH4 amplification were calculated by Bio-Rad Thermal Cyclers software. The detailed calculation was performed as follow: dCt = average Ct (HRH4)-average Ct (RNAse P); ddCt (sample1) = dCt (GC)−dCT (ANT) = 2.96; E_target_ was determined by the efficiency of target gene amplification. Cut-off value (sample1) = E^−ddCt^ = 2.05^−2.96^ = 0.12. The copy number of HRH4 in sample1 is 0.
(TIF)

Figure S2 **Prediction of potential CpG islands on the promoter of HRH4 gene (GC rate>0.6) using online tools.**
(TIF)

Figure S3 **Protein levels of HRH4 in the gastric cancer cell lines were examined using Western blot assay, and normalized with the amount of.** Shown is representative example of multiple experiments.
(TIF)

Figure S4 **FISH analysis using chromosome 18q specific alpha satellite DNA probe and chromosome 18q11 specific probe for HRH4 gene in AGS cells.**
(TIF)

Table S1 **Detailed description of Patient Characteristics in this study.**
(DOC)
