# Supplementary figures and images for: Deletion and Down-Regulation of HRH4 Gene in Gastric Carcinomas: A Potential Correlation with Tumor Progression
Source: PLoS One. 2012 Feb 20;7(2):e31207. doi: 10.1371/journal.pone.0031207 (PMC3282702; doi:10.1371/journal.pone.0031207)

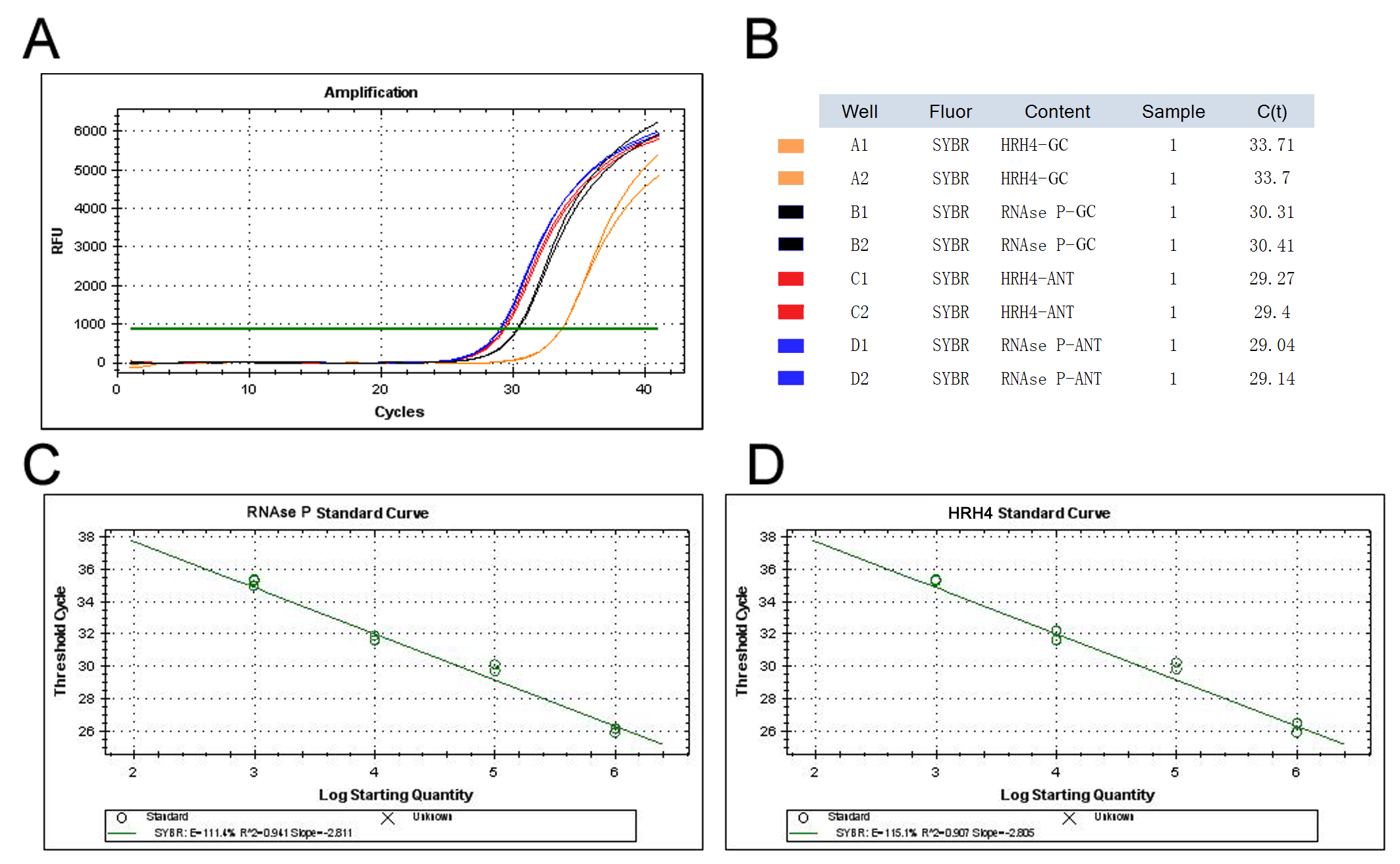

Supplement: Figure S1 — The representative diagram of the DNA CNV analysis for oneT/N pair. (A) Real-time PCR amplification of targeted gene in selected genomic DNA samples. Each data was obtained from two independent reactions. (B) Original Ct values obtained from the real-time PCR amplification. (C&D) The efficiency of and slope of the RNAse P and HRH4 amplification were calculated by Bio-Rad Thermal Cyclers software. The detailed calculation was performed as follow: dCt = average Ct (HRH4)-average Ct (RNAse P); ddCt (sample1) = dCt (GC)−dCT (ANT) = 2.96; Etarget was determined by the efficiency of target gene amplification. Cut-off value (sample1) = E−ddCt = 2.05−2.96 = 0.12. The copy number of HRH4 in sample1 is 0. (TIF) [file pone.0031207.s001.tif]

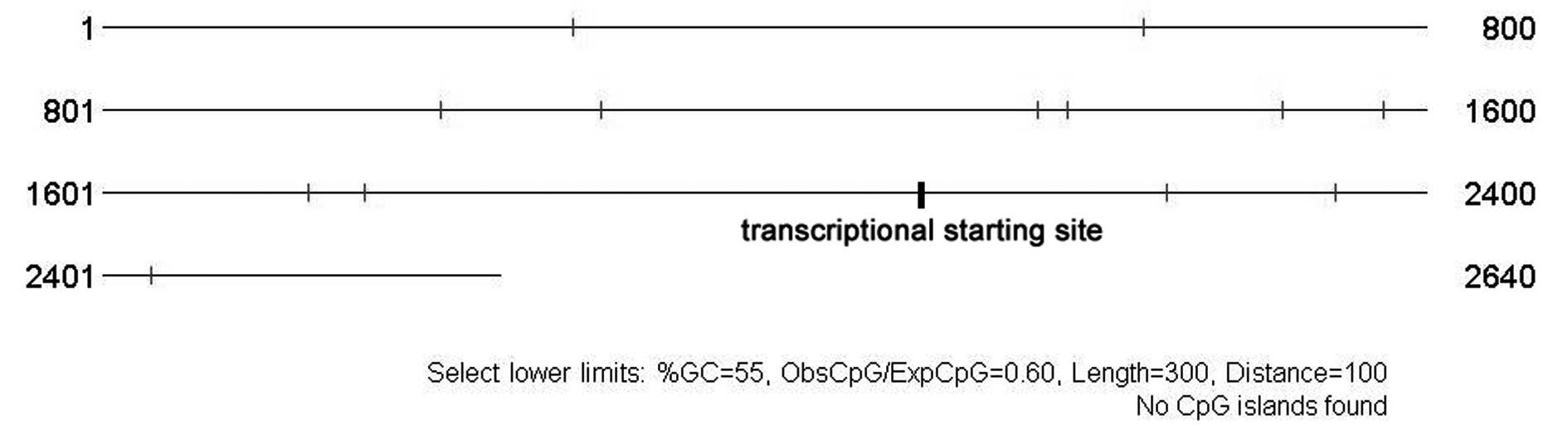

Supplement: Figure S2 — Prediction of potential CpG islands on the promoter of HRH4 gene (GC rate>0.6) using online tools. (TIF) [file pone.0031207.s002.tif]

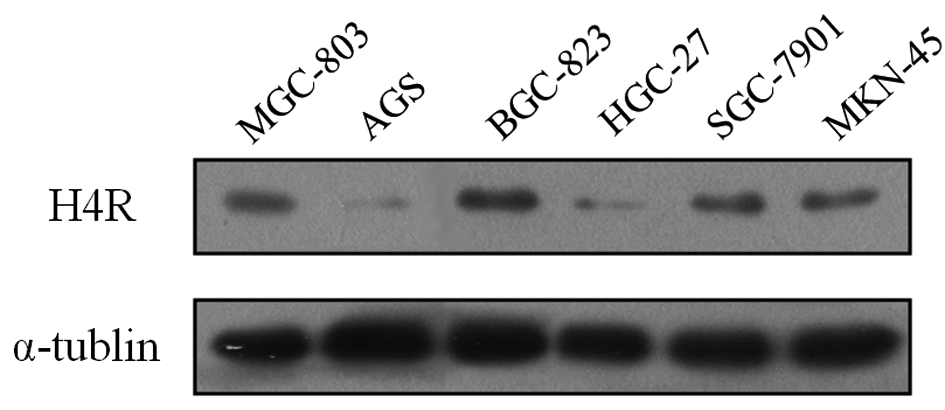

Supplement: Figure S3 — Protein levels of HRH4 in the gastric cancer cell lines were examined using Western blot assay, and normalized with the amount of. Shown is representative example of multiple experiments. (TIF) [file pone.0031207.s003.tif]

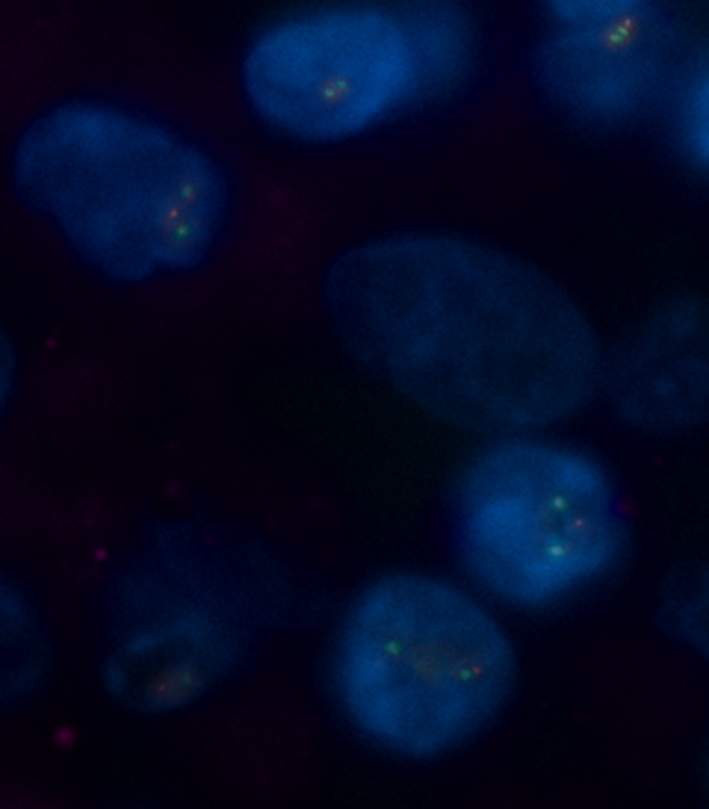

Supplement: Figure S4 — FISH analysis using chromosome 18q specific alpha satellite DNA probe and chromosome 18q11 specific probe for HRH4 gene in AGS cells. (TIF) [file pone.0031207.s004.tif]
