# Supplementary material for: Deletion and Down-Regulation of HRH4 Gene in Gastric Carcinomas: A Potential Correlation with Tumor Progression
Source: PLoS One. 2012 Feb 20;7(2):e31207. doi: 10.1371/journal.pone.0031207 (PMC3282702; doi:10.1371/journal.pone.0031207)
Supplement: Table S1 — Detailed description of Patient Characteristics in this study. (DOC) [file pone.0031207.s005.doc]

Supplementary Table S1. Detailed description of Patient Characteristics in this study

| Age (years old) | Male | Female | Smoking history(+) |
| --- | --- | --- | --- |
| 30~44 | 9 | 4 | 4 |
| 45~59 | 32 | 25 | 25 |
| 60~75 | 37 | 24 | 31 |
| Total number | 78 | 53 | 60 |
| Percentage(%) | 59.5% | 40.5% | 45.8% |
